# ﻿*Elymusmultiramosus* (Poaceae), a new species from the north-western Qinghai-Tibetan Plateau, China

**DOI:** 10.3897/phytokeys.249.127632

**Published:** 2024-11-12

**Authors:** Yong-Chao Zhang, Xiao-Xing Wei, Yan Qin, Yong Liu, Shu-Zhen Zhang, Zhi-Feng Jia, Wen-Hui Liu

**Affiliations:** 1 Laboratory for Research and Utilization of Qinghai Tibetan Plateau Germplasm Resources, Academy of Agriculture and Forestry Science of Qinghai University (Qinghai Academy of Agriculture and Forestry Sciences), Academy of Animal and Veterinary Sciences of Qinghai University (Qinghai Academy of Animal and Veterinary Sciences), Xining 810000, China Qinghai University Xining China; 2 College of Grassland Science, Xinjiang Agricultural University, Urumqi 830052, China Xinjiang Agricultural University Urumqi China

**Keywords:** Molecules, morphology, new species, phylogeny, Poaceae

## Abstract

A new species from China, *Elymusmultiramosus* Y.C. Zhang, **sp. nov.** is described and illustrated herein, based on morphological characters and molecular phylogenetic analysis. The taxonomic descriptions of *E.multiramosus* and the comparison with related species are presented. The taxonomic distinctiveness of this new species was inferred by Maximum Likelihood (ML) analysis and Bayesian phylogenetic analysis, based on the complete chloroplast genome sequence. It is assigned to the Elymus section and bears similarity to Elymusnutans Griseb. However, it can be easily distinguished from other species by its compound spike, in contrast to the simple spike inflorescence typical of those species. The compound spike is characterised by rhachillas that are extended at the base of the main axis, giving rise to 3–6 mini-spike-like branches. Notably, these branches significantly increase in length from the top towards the bottom of the compound spike. In the molecular phylogeny, *Elymusmultiramosus* from Qinghai, north-western China, is phylogenetically positioned as a distinct lineage. The lineage comprising *Elymussinosubmuticus* from Sichuan, east of the Tibetan Plateau and *Elymusnutans* from the Himalayas forms a sister group to *Elymusmultiramosus*, suggesting that these three species share a common ancestor that is distinct from the lineage leading to *Elymusatratus* from Gansu, north of the Tibetan Plateau.

## ﻿Introduction

Poaceae (grasses) is one of the largest ﬂowering plant families in angiosperms, including many economically important crops, such as rice (*Oryzasativa* L.; He et al. (2011)), barley (*Hordeumvulgare* L.; Pan et al. (2023)) and maize (*Zeamays* L.; Chen et al. (2022a) (Hodkinson 2018). Evolutionary changes in the organisation and structure of grass inﬂorescence have resulted in their different morphologies from those of core eudicots and non-grass monocots (Zanis 2007). The basic inﬂorescence unit of grasses is the spikelet, which is a short branch with leaf-like organs called glumes enclosing one or more ﬂorets. Grass plants develop distinct inﬂorescences and spikelets that determine grain yields (Gao et al. 2013). The combined number and size of seeds contribute to higher fitness in the wild and higher yield in cultivation. Accordingly, inflorescence structure and flower/seed production have been the target of both natural and human selection (Kellogg 2022).

*Elymus* L., as defined in the seminal works of Löve and Dewey (Dewey 1984; Löve 1984), stands as the most expansive genus within the Triticeae tribe, marked by its polyploid, mostly short-lived perennial grasses (Jensen and Chen 1992). Spanning about 172 species, *Elymus* extends from the Arctic to subtropical regions, adapting to diverse environments like grasslands, semi-deserts and mountainous areas. The genus’ taxonomy is complex, shaped by its morphological diversity, affected by environmental and developmental factors, polyploid origins and frequent interspecies hybridisation. Some lineages comprise many species with a wide range of ecological variation, while others encompass small groups which vary little from one another (Darwin 1951). Morphological innovations are critical for the diversiﬁcation of plants to adapt to new environments (Linder and Rudall 2005). Through the genome-wide DArTseqLD data from 57 taxa for analysis of the genetic structure and relationships within *Elymus* and some associated taxa, two major clades were identified, separating American and Eurasian species, suggesting at least two independent origins. Subclades were found within both regions and the species likely migrated multiple times between North and South America (Leo et al. 2024).

Inflorescence structure has been studied intensively in rice, maize and especially in wheat. The ‘Miracle wheat’ produces lateral meristems that sprout mini-spike-like branches predominantly from the basal part of the main axis of the spike. The mini-spike-like branches can produce their own spikelets in a distichous arrangement resulting in an indeterminate number of spikelets per spike (Poursarebani et al. 2015). In rice, mutants were created to study the inﬂorescence morphology of primary and secondary branches (Gao et al. 2013). In addition to the plant’s genetic basis, environmental factors play an important role in modification of the inflorescence. During inflorescence development, most stress response genes are highly expressed, resulting in spikelet differentiation (Li et al. 2018; Kellogg 2022).

Central Asia is an important centre of diversity for the family Poaceae (Tzvelev 1983; Nobis et al. 2020), including the genus *Elymus* L. which is the largest in the tribe Triticeae (Hodkinson 2018). The principal argument on the circumscription of *Elymus* is whether there are single or multiple spikelets per rachis node (Sun and Salomon 2009). In the description of *Elymus* L. in the web of Plants of the World Online, the inflorescence of *Elymus* L. is either composed of racemes, fertile sessile spikelets and with a barren rhachilla extension or with diminished florets at the apex.

*Elymus* provides important perennial forage in temperate regions of the world and especially in the Qinghai-Tibetan Plateau. In October 2020, in the city of Delingha, west of the Qinghai-Tibetan Plateau, a plant with a compound spike was discovered in a dry, rocky area of alpine meadow. This plant appeared to represent a new species of the genus *Elymus* L. This species occurs in the central region of the north-western margin of the Tibetan Plateau, defined by the Kunlun Mountains and is characterised by high altitudes, low temperatures and minimal precipitation. The eastern segment of the Kunlun Mountains receives more precipitation, ranging from 300 to 600 mm annually, while the central and western segments are much drier. Most of the region experiences average annual temperatures below 0 °C, with particularly harsh winters. These extreme climatic conditions have intensified ecological pressures, resulting in unique vegetation and ecosystems. Multiple glaciation events during the Quaternary Period have further shaped the region’s geomorphology and water resources. The Kunlun Mountains’ ecosystems, formed since the Pliocene, feature distinctive vegetation types, including high-altitude grasslands, temperate deciduous forests and glacier-adapted vegetation, with marked differences between the southern and northern slopes due to climatic variations (Du 2021).

The aim of this paper is to describe and classify this newly-discovered species, distinguished by its unique compound spike. The study involves comparing this species with existing species within the genus, particularly *Elymusnutans* and providing molecular evidence to confirm its status as a distinct species. Additionally, the research seeks to introduce the compound spike as a significant characteristic for the classification within the genus *Elymus*.

## ﻿Materials and methods

### ﻿Plant materials

The new species *Elymusmultiramosus* described below, is known only from a small area of Delingha City, west of Qinghai Province (37°29'14"N, 97°23'27"E). Presently, only five populations with approximately 26 individuals had been found. Every individual of this new species possessed over 60 tillers and each tiller featured a compound spike. Morphological observations and dissections of this new species were made under a stereoscopic microscope (Nikon SMZ18, Tokyo, Japan). Karyotype analysis was carried out and compared with two germplasm of *Elymussibiricus* L. with the sample numbers 15–262 and 16–118 provided by Qinghai Academy of Animal Science and Veterinary Medicine, Qinghai University, China.

### ﻿Chloroplast sequencing and genome annotation

DNA extraction was performed by utilising the TianGen CTAB method kit. Following the DNA extraction, we prepared the sequencing library, ensuring its integrity through PCR amplification and subsequent quality assessment. Once the library met the quality standards, it underwent sequencing on the Illumina NovaSeq platform and employed 150 bp pair-end (PE) read length for sequencing, ensuring adequate coverage depth and accurate sequence information. The sequence data were processed using GetOrganelle1.7.5 software. Finally, genome annotation was performed using the Geneious and Geseq software. The data reported in this paper have been deposited in the GenBase at the National Genomics Data Center (Members and Partners 2023), Beijing Institute of Genomics, Chinese Academy of Sciences/China National Center for Bioinformation, under accession number C_AA070531 that is publicly accessible at https://ngdc.cncb.ac.cn/genbase.

### ﻿Phylogenetic analysis

To conduct this study, the chloroplast genome was selected for phylogenetic analysis, with the removal of inverted repeat regions to enhance the accuracy of the analysis. Sequence alignment was then performed using the MAFFT software on these selected regions. To improve alignment quality, Gblocks software was employed to extract highly-conserved regions, which are more suitable for phylogenetic inference. Following alignment, the ModelFinder tool was utilised to identify the best-fit substitution model for the dataset. The GTR+F+R3 model was selected as the optimal choice for the analysis (Nguyen et al. 2015). Based on the selected substitution model, the phylogenetic tree was constructed using IQ-TREE software. To ensure robustness and reliability, Bayesian Inference (MrBayes) was also used to construct a phylogenetic tree. The trees generated by the Maximum Likelihood method (IQ-TREE) and Bayesian Inference (MrBayes) were compared, providing comprehensive validation of the consistency and robustness of the phylogenetic inferences.

To determine the phylogenetic position of *Elymusmultiramosus*, the complete cp genome sequences of 39 species were analysed. These included 36 species from Poaceae family and three species from Solanaceae Juss., Brassicaceae Burnett and Fabaceae Lindl. families, which served as the outgroup. Amongst the 36 Poaceae species, 24 belonged to *Elymus* genus, three to the *Campeiostachys* Drobow, two to Hordeum L., one species each from *Pseudoroegneria* (Nevski) Á. Löve, *Triticum* L., *Thinopyrum* Á. Löve, *Aegilops* L., *Secale* L., *Agropyron* Gaertn., *Thinopyrum* Á.Löve and *Brachypodium* P. Beauv. Table 1 provides detailed information on the species and their corresponding GenBank accession numbers.

**Table 1.** Basic information on species involved in Phylogenetic analysis.

Family	Genus	Species	Accession number
Poaceae	*Elymus*	*Elymusciliaris* (Trin.) Tzvelev	MK775252.1
Poaceae	*Elymus*	*Elymussubmuticus* (Keng) Á.Löve	MT644143.1
Poaceae	*Elymus*	*Elymusrepens* (L.) Gould	NC_058753.1
Poaceae	*Elymus*	*Elymusgrandis* (Keng) S. L. Chen	MN703669.1
Poaceae	*Elymus*	Elymusnodosussubsp.caespitosus (K.Koch) Melderis	MK775251.1
Poaceae	*Elymus*	*Elymuskamoji* (Ohwi) S. L. Chen	NC_051511.1
Poaceae	*Elymus*	*Elymustauri* (Boiss. & Balansa) Melderis	MT385864.1
Poaceae	*Elymus*	*Elymussibiricus* L.	MK775250.1
Poaceae	*Elymus*	*Elymusgmelinii* (Ledeb.) Tzvelev	NC_066043.1
Poaceae	*Elymus*	*Elymusbreviaristatus* (Keng) Keng f.	MT644142.1
Poaceae	*Elymus*	*Elymustrachycaulus* (Link) Gould ex Shinners	MW752517.1
Poaceae	*Elymus*	*Elymussinosubmuticus* S. L. Chen	MT644146.1
Poaceae	*Elymus*	*Elymuspendulinus* (Nevski) Tzvelev	NC_066045.1
Poaceae	*Elymus*	*Elymusstrictus* (Keng) S. L. Chen	MZ736600.1
Poaceae	*Elymus*	*Elymusalashanicus* (Keng) S. L. Chen	OL444890.1
Poaceae	*Elymus*	*Elymushystrix* L.	NC_058749.1
Poaceae	*Elymus*	*Elymuscognatus* (Hack.) T. A. Cope	MT385860.1
Poaceae	*Elymus*	*Elymuslibanoticus* (Hack.) Melderis	MT385861.1
Poaceae	*Elymus*	*Elymusstipifolius* (Trautv.) Melderis	MT385862.1
Poaceae	*Elymus*	*Elymusnutans* Griseb.	NC_058918.1
Poaceae	*Elymus*	*Elymusatratus* (Nevski) Hand.-Mazz.	MT610373.1
Poaceae	*Elymus*	*Elymuslongearistatus* (Boiss.) Tzvelev	MN703670.1
Poaceae	*Elymus*	*Elymusvirginicus* L.	NC_058750.1
Poaceae	*Elymus*	*Elymusmagellanicus* (É.Desv.) Á.Löve	MZ337548.1
Poaceae	*Campeiostachys*	*Campeiostachyskamoji* (Ohwi) B.R.Baum, J.L.Yang & C.Yen	MW043483.1
Poaceae	*Campeiostachys*	*Campeiostachysdahurica* (Turcz. ex Griseb.) B.R.Baum, J.L.Yang & C.Yen	NC_049159.1
Poaceae	*Campeiostachys*	Campeiostachysdahuricavar.tangutorum	MN420499.1
Poaceae	*Thinopyrum*	*Thinopyrumelongatum* (Host) D.R.Dewey	MW888707.1
Poaceae	*Pseudoroegneria*	*Pseudoroegneriaspicata* (Pursh) Á.Löve	MH285855.1
Poaceae	*Triticum*	*Triticumaestivum* L.	KJ614396.1
Poaceae	*Aegilops*	Aegilopsspeltoidesvar.speltoides	KJ614406.1
Poaceae	*Secale*	*Secalecereale* L.	KC912691.1
Poaceae	*Agropyron*	*Agropyroncristatum* L.	MN703653.1
Poaceae	*Hordeum*	Hordeumvulgaresubsp.vulgare	NC_008590.1
Poaceae	*Hordeum*	*Hordeumbogdanii* Wilensky	NC_043839.1
Poaceae	*Brachypodium*	*Brachypodiumdistachyon* (L.) P.Beauv.	NC_011032.1
Solanaceae	*Solanum*	*Solanumtuberosum* L.	NC_008096.2
Brassicaceae	*Arabidopsis*	*Arabidopsisthaliana* (L.) Heynh.	AP000423.1
Fabaceae	*Melilotus*	*Melilotusalbus* Medik.	NC_041419.1

## ﻿Results

Specimens of the potential new species were collected in Baishu Mountain, Delhi City, Qinghai, China. Morphological comparisons between the new species and its morphologically similar species of the genus *Elymus* were found in the Flora Reipublicae Popularis Sinicae (https://www.iplant.cn/frps) and the Flora Qinghaiica. Diagnostic characters involved in inflorescence morphology, spikelet number and glume, palea apex and lemma, leaf sheath and leaf characteristics, culm characteristics are shown in Tables 2–6. Morphological descriptions mainly referred to the Flora Reipublicae Popularis Sinicae.

**Table 2.** Morphological inflorescence comparison of *Elymusmultiramosus* with related *Elymus* species.

Species	Inflorescence					
	Type	Tightness	Upright or Bent	Total length	Branch number	Branch length
*Elymusmultiramosus* Y.C. Zhang	compound spike	slightly lax	pendulous	17–19	3–6	2–4
*Elymusbreviaristatus* (Keng) Keng f.	spike	lax	tender and pendulous	10–15	no	no
*Elymussinosubmuticus* S. L. Chen	spike	laxer	curved	3.5–7.5	no	no
*Elymussibiricus* L.	spike	laxer	pendulous	15–20	no	no
*Elymusatratus* (Nevski) Hand.-Mazz.	spike	denser	flexuous, pendulous	5–8	no	no
*Elymusnutans* Griseb.	spike	denser	flexuous, apex pendulous	5–12	no	no
*Elymuscanadensis* L.	spike	denser	pendulous	12–20	no	no
*Elymusvillifer* C. P. Wang & H. L. Yang	spike	denser	slightly curved	9–12	no	no
*Elymuspurpuraristatus* C. P. Wang & H. L. Yang	spike	denser	erect or slightly curved	8–15	no	no
*Elymusdahuricus* Turcz. var. violeus C. P. Wang & H. L. Yang	spike	denser	curved	18.5–25.5	no	no
ElymusdahuricusTurcz.var.dahuricus	spike	denser	erect	14–18	no	no
*Elymusexcelsus* Turcz.	spike	laxer	erect	15–22	no	no
Elymusdahuricusvar.cylindricus Franch.	spike	denser	erect	7–14	no	no
*Elymustangutorum* (Nevski) Hand.-Mazz.	spike	denser	erect	8–15	no	no
*Elymusdahuricus* Turcz.	spike	denser	erect	14–18	no	no
*Elymusbarystachyus* L. B. Cai	spike	denser	erect	8–18	no	no

**Table 3.** Morphological comparisons of spikelet number and glume comparison of *Elymusmultiramosus* with related *Elymus* species.

Species	Spikelet number	Glume				
		Type	Length (mm)	Veins number	Apex type	Apex awn length (mm)
*Elymusmultiramosus* Y.C. Zhang	1–2	Lanceolate	4–7	3	awned	1.5–2.2
*Elymusbreviaristatus* (Keng) Keng f.	2	oblong or ovate lanceolate	3–4	1–3	acuminate or mucro	1
*Elymussinosubmuticus* S. L. Chen	1–2	oblong	2–3	3	acute or acuminate	no cuspidate
*Elymussibiricus* L.	1–2	Narrowly lanceolate	4–5	3–5	acuminate or a short awn	4
*Elymusatratus* (Nevski) Hand.-Mazz.	2	Narrowly oblong or lanceolate	2–4	1–3	acuminate	< 1
*Elymusnutans* Griseb.	1–2	oblong	3–4	3–4	acuminate or a short awn	1–4
*Elymuscanadensis* L.	2–3	Linear	3–4	3–4	awned	7–18
*Elymusvillifer* C. P. Wang & H. L. Yang	1–2	Narrowly lanceolate	4.5–7.5	3–4	acuminate to an awned tip	1.5–2.5
*Elymuspurpuraristatus* C. P. Wang & H. L. Yang	2	Lanceolate to linear-lanceolate	7–10	3	mucro	1
*Elymusdahuricus* Turcz. var. violeus C. P. Wang & H. L. Yang	1–2	Lanceolate	7–11	3–5	awned	3–6
*ElymusdahuricusTurcz.var.dahuricus*	1–2	Lanceolate or linear-lanceolate	8–10	3–5	awned	5
*Elymusexcelsus* Turcz.	2–4	Narrowly lanceolate	10–13	5–7	awned	7
Elymusdahuricusvar.cylindricus Franch.	1–2	Lanceolate to linear-lanceolate	7–8	3–5	acuminate	4
*Elymustangutorum* (Nevski) Hand.-Mazz.	1–2	Lanceolate to linear-lanceolate	7–10	5	acuminate	1–3
*Elymusdahuricus* Turcz.	1–2	Lanceolate to linear-lanceolate	7–10	3–5	acuminate or awned	5
*Elymusbarystachyus* L. B. Cai	2	linear-lanceolate	7–10	4–7	acuminate or pointed	1.5

**Table 4.** Morphological comparisons of palea apex and lemmas of *Elymusmultiramosus* with related *Elymus* species.

Species	Palea apex	Lemmas			
		Type	Vein number	First lemmas length (mm)	Awn length (mm)
*Elymusmultiramosus* Y.C. Zhang	rounded or flattened	lanceolate	3	7–10	9–12
*Elymusbreviaristatus* (Keng) Keng f.	obtuse-rounded or slightly concave	lanceolate	5	8–9	1–5
*Elymussinosubmuticus* S. L. Chen	obtuse-rounded	lanceolate	5	7–8	2
*Elymussibiricus* L.	2-lobed	lanceolate	5	8–11	10–15
*Elymusatratus* (Nevski) Hand.-Mazz.	obtuse-rounded	lanceolate	5	7–8	10–17
*Elymusnutans* Griseb.	obtuse-rounded or truncate	oblong-lanceolate	5	10	12–20
*Elymuscanadensis* L.	pointed or obtusely rounded and retuse	lanceolate	5	10–17	20–30
*Elymusvillifer* C. P. Wang & H. L. Yang	–	oblong-lanceolate	5	7–11	
*Elymuspurpuraristatus* C. P. Wang & H. L. Yang	–	oblong-lanceolate		6–9	7–15
*Elymusdahuricus* Turcz. var. violeus C. P. Wang & H. L. Yang	–	lanceolate		9–21	9–21
ElymusdahuricusTurcz.var.dahuricus	truncate	lanceolate	5	9	10–20
*Elymusexcelsus* Turcz.	–		5	8–12	15–40
Elymusdahuricusvar.cylindricus Franch.	obtuse-rounded	lanceolate	5	7–8	6–13
*Elymustangutorum* (Nevski) Hand.-Mazz.	obtuse-headed	lanceolate	5	8–12	3–11
*Elymusdahuricus* Turcz.	narrowly truncate	lanceolate		7–9	2–20
*Elymusbarystachyus* L. B. Cai	–	oblong-lanceolate		7–8	1–2

**Table 5.** Morphological comparisons of leaf sheath and leaf of *Elymusmultiramosus* with related *Elymus* species.

Species	Leaf-sheath type	Leaf type	Leaf length (cm)	Leaf width (mm)
*Elymusmultiramosus* Y.C. Zhang	glabrous	blade flattened	18–22	5–7
*Elymusbreviaristatus* (Keng) Keng f.	glabrous	blade flattened	4–12	3–5
*Elymussinosubmuticus* S. L. Chen	glabrous	blade flattened or involute	3–6	1.5–3
*Elymussibiricus* L.	smooth and glabrous	blade flattened	10–20	5–10
*Elymusatratus* (Nevski) Hand.-Mazz.	smooth and glabrous	blade or involute	3–10	2
*Elymusnutans* Griseb.	glabrous	blade flattened,sparsely pilose above, scabrous or smooth below	6–8	3–5
*Elymuscanadensis* L.	glabrous	blade flattened	20–30	7–15
*Elymusvillifer* C. P. Wang & H. L. Yang	densely villous	flattened or margins involute	9–15	3–6
*Elymuspurpuraristatus* C. P. Wang & H. L. Yang	glabrous	blades often involute	15–25	2.5–4
*Elymusdahuricus* Turcz. var. violeus C. P. Wang & H. L. Yang	base densely white villous	blade flattened or drying involute	20–35	8.7–13.6
ElymusdahuricusTurcz.var.dahuricus	smooth and glabrous	blade flattened, sparsely involute	15–25	5–12
*Elymusexcelsus* Turcz.	glabrous	flattened	20–30	10–16
Elymusdahuricusvar.cylindricus Franch.	glabrous	blade flattened	5–12	5
*Elymustangutorum* (Nevski) Hand.-Mazz.	smooth	blade flattened	10–20	6–14
*Elymusdahuricus* Turcz.	glabrous, or densely pilose at base	blade glaucous, flat, rarely rolled	5–25	5–12
*Elymusbarystachyus* L. B. Cai	glabrous	blade glabrous on both surfaces	7–22	4–8

**Table 6.** Morphological comparisons of culm of *Elymusmultiramosus* with related *Elymus* species.

Species	Culms bushy type	Culms type	Culms height (cm)
*Elymusmultiramosus* Y.C. Zhang	tufted	erect, base slightly decumbent	82–95
*Elymusbreviaristatus* (Keng) Keng f.	sparsely tufted	erect or basally geniculate, short, decurrent rhizomes	70
*Elymussinosubmuticus* S. L. Chen	tufted	erect or base slightly geniculate, weak	25–45
*Elymussibiricus* L.	solitary or sparsely tufted	erect or base slightly inclined	60–90
*Elymusatratus* (Nevski) Hand.-Mazz.	sparsely tufted	erect, weak	40–60
*Elymusnutans* Griseb.	tufted	erect, base slightly geniculate	50–70
*Elymuscanadensis* L.	few tufted	erect or base slightly geniculate	100
*Elymusvillifer* C. P. Wang & H. L. Yang	sparsely tufted	erect	60–75
*Elymuspurpuraristatus* C. P. Wang & H. L. Yang	tufted	erect, stout	160
*Elymusdahuricus* Turcz. var. violeus C. P. Wang & H. L. Yang	sparsely tufted	erect	145–225
ElymusdahuricusTurcz.var.dahuricus	sparsely tufted	erect	70–140
*Elymusexcelsus* Turcz.	tufted	erect, robust	140
Elymusdahuricusvar.cylindricus Franch.	tufted	erect, weak	40–80
*Elymustangutorum* (Nevski) Hand.-Mazz.	tufted	erect, tall and stout, base geniculate	120
*Elymusdahuricus* Turcz.	tufted	erect, base geniculate	40–140
*Elymusbarystachyus* L. B. Cai	laxly tufted or solitary	erect, base geniculate	50–80

*Elymusmultiramosus* is distinguished by its compound spike, which is slightly lax and pendulous, measuring 17–19 cm in total length, with 3–6 branches, each 2–4 cm long. This contrasts sharply with the other species, which predominantly exhibit a simple spike with no branches. For instance, *Elymusbreviaristatus* has a spike that is lax, but unbranched and *Elymussibiricus* shows a similarly unbranched lax spike. In addition, *Elymusexcelsus* has a spike of comparable length (15–22 cm), but it also lacks the branched structure that characterises *Elymusmultiramosus*, further emphasising the unique morphological traits of the latter (Table 2). In terms of spikelet number, *Elymusmultiramosus* typically bears 1–2 spikelets per node, which is similar to species such as *Elymussinosubmuticus* and *Elymussibiricus*. However, the glume morphology sets *Elymusmultiramosus* apart, as its glumes are lanceolate, 4–7 mm in length, with three veins and bear awns of 1.5–2.2 mm. In contrast, *Elymuscanadensis* features linear glumes with significantly longer awns (7–18 mm). Additionally, *Elymusrosthornii* and *Elymusvillifer* exhibit distinct glume shapes and awn lengths, highlighting further differences amongst these species (Table 3). The palea apex of *Elymusmultiramosus* is rounded or flattened, while its lemmas are lanceolate with three veins, the first lemmas measuring 7–10 mm in length and bearing awns 9–12 mm long. Other species, such as *Elymussibiricus* and *Elymuscanadensis*, have longer lemma awns, reaching up to 20–30 mm, much longer than those of *Elymusmultiramosus*. Additionally, *Elymusnutans* has truncate palea apices, with awns measuring 12–20 mm, creating a notable morphological distinction from *Elymusmultiramosus*. These differences play a critical role in distinguishing species within this genus (Table 4). *Elymusmultiramosus* has glabrous leaf sheaths and flattened blades, with leaves measuring 18–22 cm in length and 5–7 mm in width. This contrasts with species like *Elymusbreviaristatus* and *Elymussinosubmuticus*, which have shorter and narrower leaves. For instance, *Elymusvillifer* has leaves 9–15 cm long and 3–6 mm wide and their margins may be involute, unlike the consistently flattened leaves of *Elymusmultiramosus*. Additionally, *Elymuspurpuraristatus* has even narrower leaves, measuring only 2.5–4 mm in width. These morphological differences help in identifying and differentiating species within the group (Table 5). The culms of *Elymusmultiramosus* are tufted, with an erect base that is slightly decumbent and they range in height from 82 to 95 cm. This is contrasted with the culms of *Elymuscanadensis*, which are taller, reaching up to 100 cm and have a more erect and less decumbent base. *Elymuspurpuraristatus*, with culms up to 160 cm, far exceeds the height of *Elymusmultiramosus*, showcasing the range of variation in culm height across species. *Elymussibiricus*, on the other hand, has shorter culms (60–90 cm) with a more inclined base, creating a stark difference in growth habit when compared to *Elymusmultiramosus* (Table 6).

### ﻿Taxonomic treatment

#### ﻿ Elymus multiramosus

Taxon classificationPlantaePoalesPoaceae

Y.C.Zhang sp. nov.

7199B7A4-B75D-5355-AF0E-71281A99F54B

urn:lsid:ipni.org:names:77351702-1

Figs 1 , 2 , 3

##### Type.

China • Qinghai, Delhi City, Baishu Mountain. 37°29.23N, 97°23.45′E, 3722 m a.s.l., 10 October 2020, *Yong-Chao Zhang* (holotype at Qinghai-Tibetan Plateau Museum of Biology, HNWP 371720).

**Figure 1.**
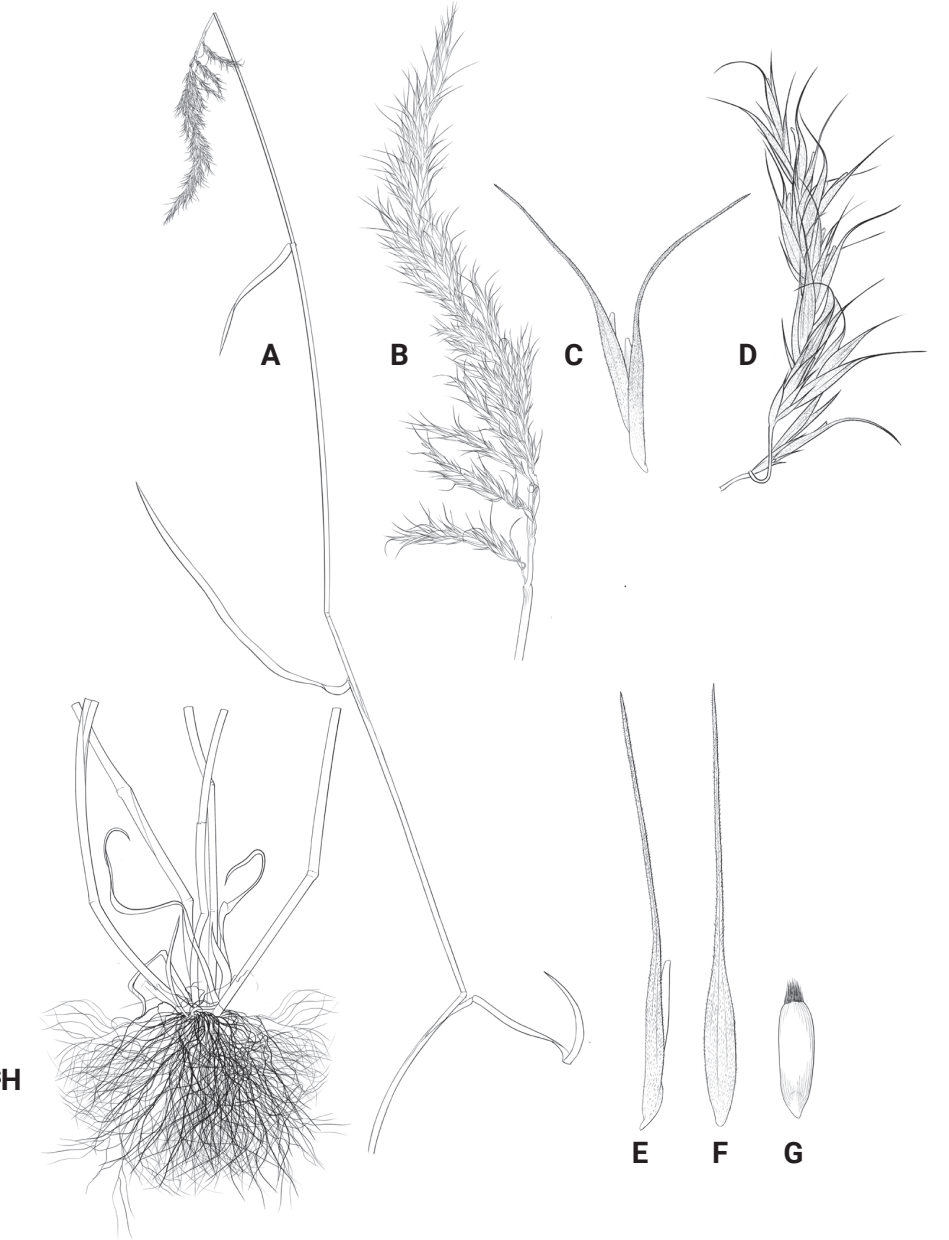
Illumination of *Elymusmultiramosus* Y.C.Zhang, sp. nov. **A** habit **B** compound spike **C** spikelet **D** primary branch **E, F** lemma **G** seed **H** base of plant with a fibrous root. Drawn by Yongchao Zhang.

##### Diagnosis.

Excluding the compound spike characteristic, *Elymusmultiramosus* shares notable similarities with *Elymussibiricus* in several traits. Both species have 1–2 spikelets per node, comparable glume lengths and vein numbers and similar lemma awn lengths. Their leaf morphology is also quite alike, with glabrous sheaths and comparable leaf blade dimensions. Additionally, their culm heights overlap, as both species exhibit tufted, erect culms, further highlighting their morphological resemblance. Similarly, *Elymusmultiramosus* closely resembles *Elymusnutans*, especially in terms of spikelet number and glume features. Both species have 1–2 spikelets per node and nearly identical lemma awn lengths, though *Elymusnutans* tends to have slightly longer awns and an oblong-shaped glume (Hua 2007). Their leaf blades are also alike, being smooth or nearly smooth, with similar dimensions, further emphasising the parallels between these two species. Lastly, Elymusdahuricusvar.dahuricus and *Elymusmultiramosus* display significant similarity in leaf morphology. Both species have glabrous sheaths and flattened leaf blades with matching lengths and widths. Their culm height ranges overlap considerably and both have tufted, erect culms. Despite these similarities, the subtle differences in their overall structure allow for their differentiation.

**Figure 2.**
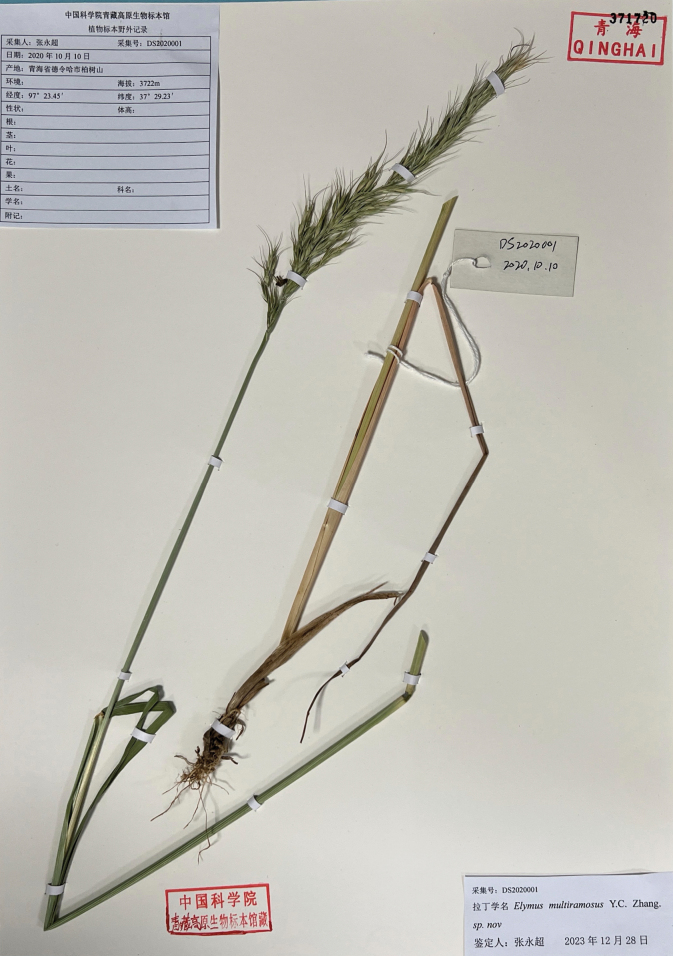
One holotype specimen of *Elymusmultiramosus* (371720), collected and preserved at Qinghai-Tibetan Plateau Museum of Biology (HNWP).

*Elymus* species are characterised by their perennial, tufted growth, typically lacking rhizomes and erect culms, sometimes decumbent at the base, with leaf sheaths split to the base. The leaves are linear or lanceolate, either flat or rolled, with membranous, non-ciliate ligules. The inflorescence is a spike, either erect or nodding, with 1–2 laterally compressed spikelets per node, each containing 2–10 florets that disarticulate below the fertile floret at maturity. The glumes are linear-lanceolate with 1–9 veins, often awned, and the lemmas are 5-veined, rounded on the back and typically awned at the apex. The caryopsis fruit adheres to both the lemma and palea (Hua 2007).

**Figure 3.**
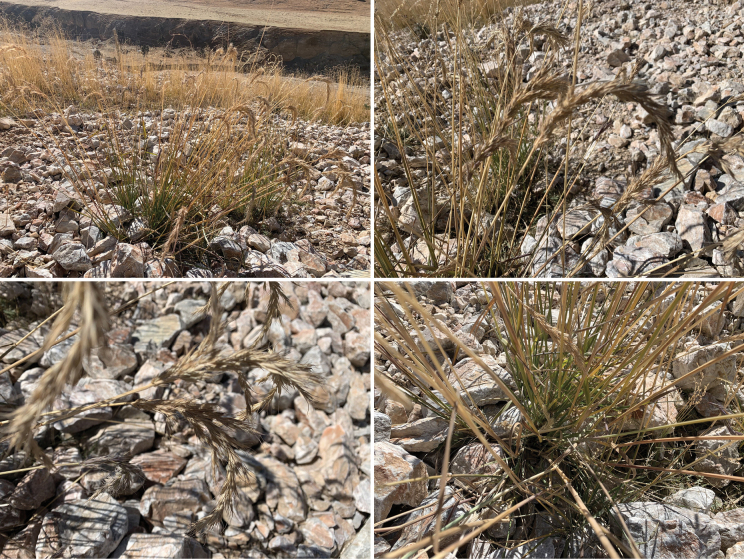
*Elymusmultiramosus* in the wild, the top left is the locality found; the top right and bottom left are the compound spikes; the bottom right is the stem and leaf. Photographs by Yongchao Zhang.

**Figure 4.**
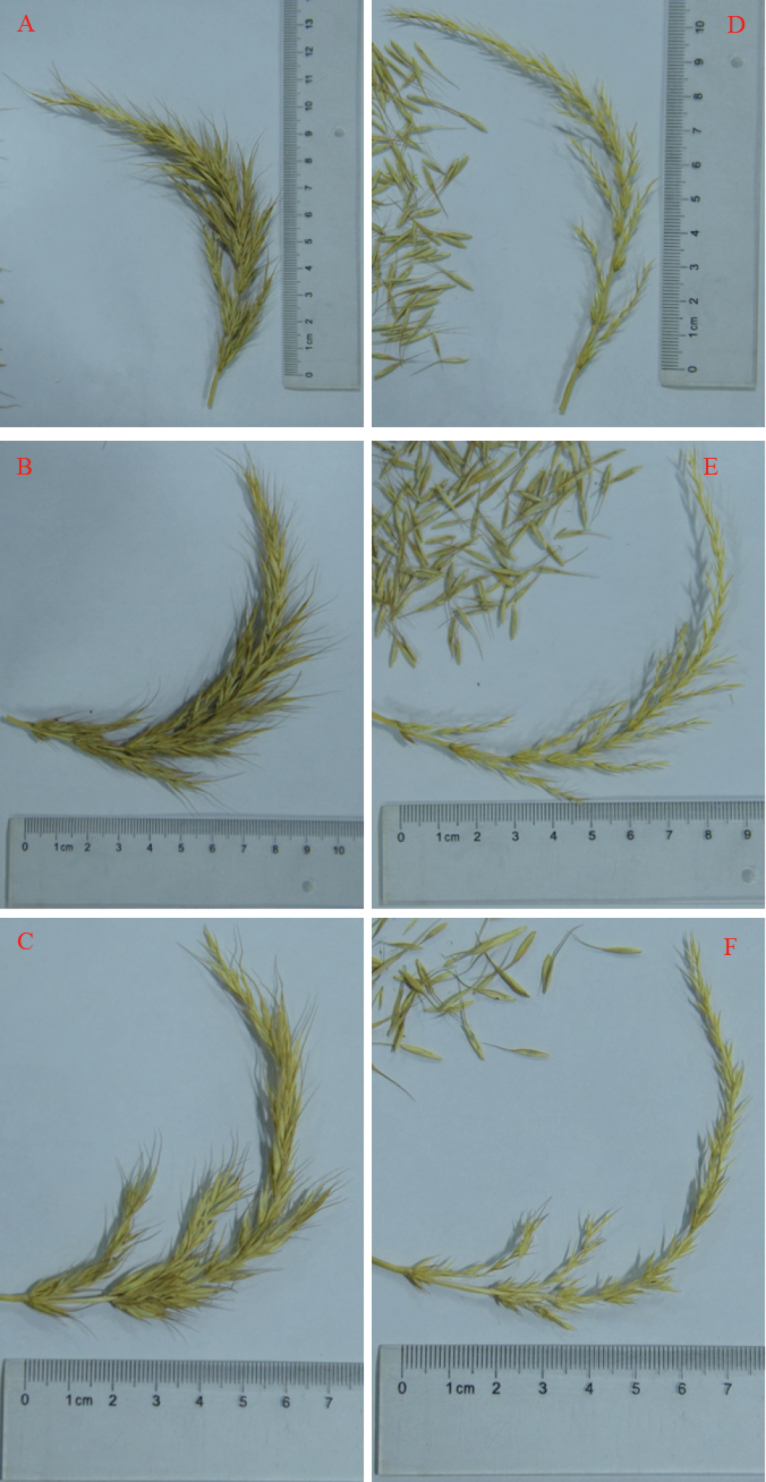
The compound spike of *Elymusmultiramosus***A–C** are three compound spikes from *Elymusmultiramosus* and **D–F** are compound spikes after seed threshing of the **A–C** separately, the mini-spike-like branches can be seen.

**Figure 5.**
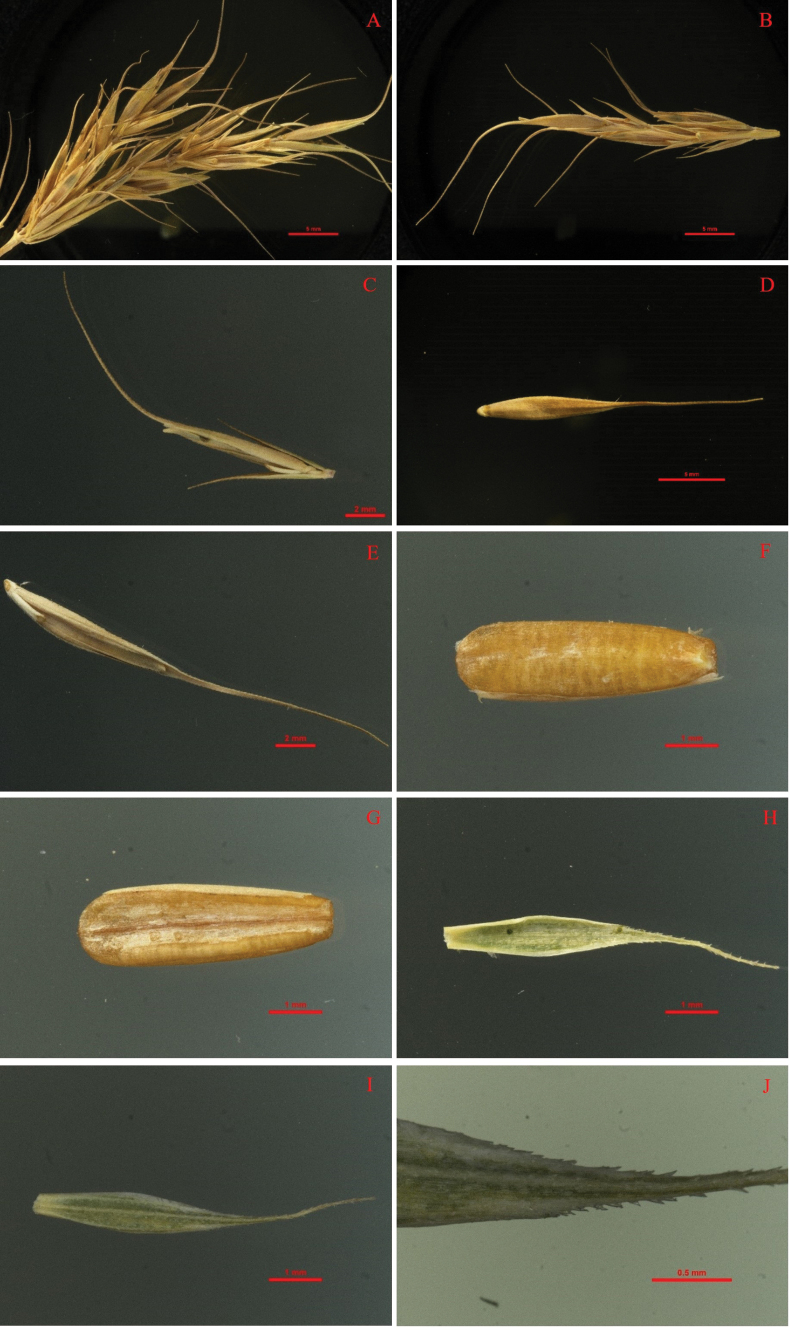
Detail spikelet of *Elymusmultiramosus* during the mature stage **A** part of compound spike with a branch **B** branch **C** spikelet **D** lemma **E** glumelle **F** seed back **G** seed ventral **H** glume ventral **I** glume back **J** glume awn and vein.

**Figure 6.**
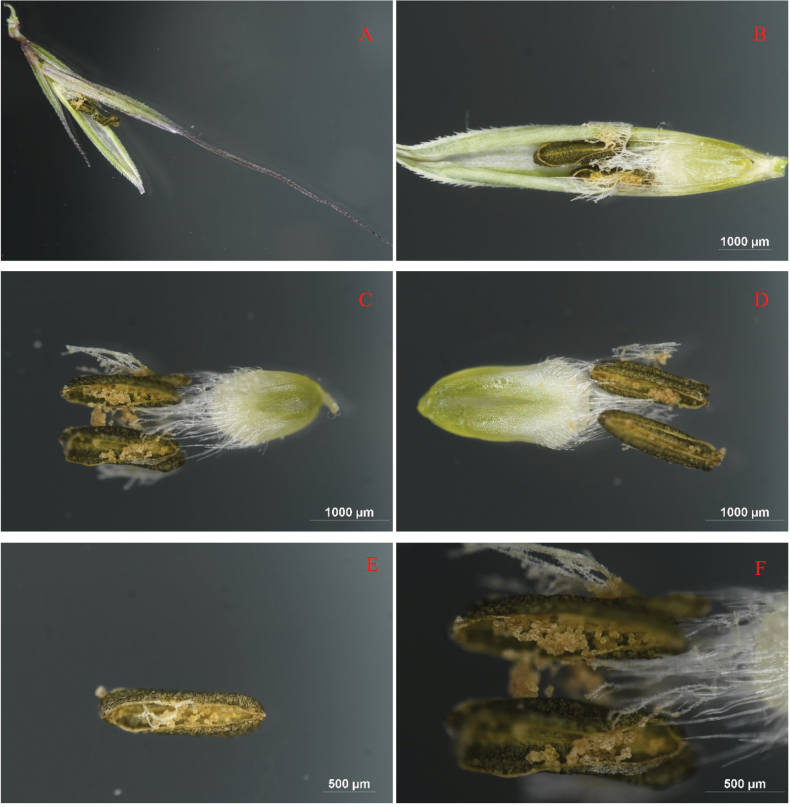
Details of compound spike of *Elymusmultiramosus* during the flowering stage **A** spikelet **B** flower **C, D** stamens, pistil, anthers **E** anther **F** pollen, stigmas.

The new species is similar to *Elymus* sp. but it can be easily distinguished from that species by its compound spike, which has 3–4 spikelets with extended rhachillas at the base nodes. These rhachillas become much longer from the top to the base of the compound spike (Figs 4–6). The glumes of *Elymusmultiramosus* are noticeably shorter than the first floret. The awns at the lemma apex range in length from 9 to 12 mm, surpassing the length of the lemma body. *Elymusmultiramosus* is distinguished by stouter plants and longer inflorescences, which measure 17 to 19 cm, with spikelets primarily arranged on one side of the rachis. The glume apices are awn-tipped and the glumes themselves are lanceolate, exceeding the length of those in *Elymusnutans* by 4 to 7 mm.

##### Description.

Culms are usually erect, sometimes slightly decumbent at base, 82–95 cm tall. Leaf-sheath glabrous; leaf blade flat, 18–22 × 0.5–0.7 cm, glabrous or adaxial surface slightly pubescent. Compound spike pendulous, slightly lax, 17–19 cm; rachis margin scabrous, no ciliolate, rachis knot dilated. Compound spike includes a clear main shoot axis and a series of lateral branches produced by the main shoot. Flower formed from the top of the main axis and primary branches from the base to the middle of the main axis. A total of 3–6 primary branches are formed in the main axis, 2–4 cm length. Each primary branch has 3–7 nodes. Spikelet usually 2 per node, with 2 or 3 florets. Glumes lanceolate, 4–7 mm, 3-veined, glabrous, scabrous along veins, apex with awn 1.5–2.2 mm. Lemma lanceolate, 3-veined, obscurely at the base, scabrous or puberulent at the apex and edge; first lemma 7–10 mm; awn 9–12 mm. Palea equalling lemma, ciliolate along keels, puberulent between keels.

##### Phenology.

*Elymusmultiramosus* flowers in early September and bears fruit in early October.

##### Etymology.

The specific epithet multiramosus is a compound of the Latin words multi meaning many and ramosus meaning branches to indicate a specific type of inflorescence.

##### Vernacular name.

Duō Zhī Pī Jiǎn Cǎo (Chinese pronunciation); 多支披碱草 (Chinese name).

##### Distribution and habitat.

The species is presently known only from a small area of Delingha City, west of Qinghai Province (37°29′14"N, 97°23′27"E). It grows on a dry rocky area of alpine, at an elevation of 3722 m a.s.l. Other plants in the vicinity of the plant include *Juniperusprzewalskii* Kom., *Agropyroncristatum* J.Gaert.,ElymusdahuricusTurcz.var.cylindricus Franchet, *Neotriniasplendens* (Trin.) M. Nobis, P. D. Gudkova & A. Nowak etc.

### ﻿Karyotype analysis

**Figure 7.**
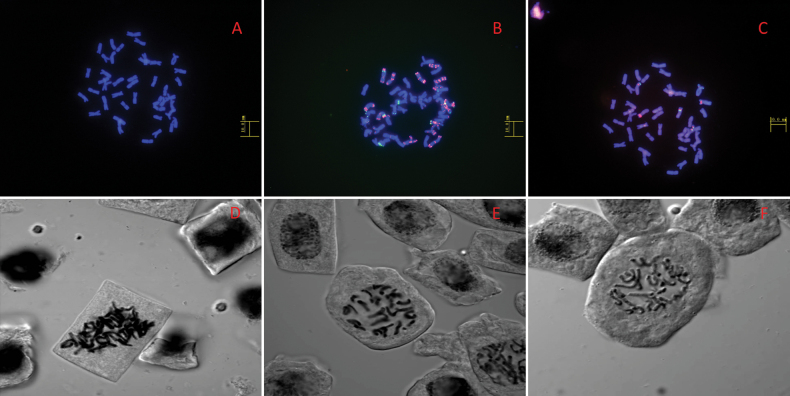
Chromosome fluorescence staining with DAPI *Elymusmultiramosus* (**A**). Chromosome repeat fluorescence in situ hybridisation using Oligo-pSc119.2 (green) and Olig-pTa535 (red) probe of *Elymusmultiramosus* (**B**). rDNA fluorescence in situ hybridisation of *Elymusmultiramosus* chromosomes, 5SrDNA is red and 18SrDNA is green (**C**). Root tip chromosomal tableting, from left to right is *Elymusmultiramosus* (2n = 6x = 42) (**D**). *Elymussibiricus* 16-118 (2n = 4x = 28) (**E**). *Elymussibiricus*15-262 (2n = 4x = 28) (**F**).

A total of 42 chromosomes were obtained by DAPI fluorescence staining, with a length of 5.0–8.5 μm, mainly proximal middle and proximal centromeres, the end of the chromosome being rich in heterochromatin and the genome is large (Fig. 7A).

In situ fluorescence hybridisation using Oligo-pSc119.2 and Olig-pTa535 probes shown that 14 chromosomes have a strong Olig-pTa535 signal at the end and possibly belong to H chromosome group (Fig. 7B).

5SrDNA and 18SrDNA repeat probes were used for fluorescence in situ hybridisation and it was found that six chromosomes had 5SrDNA hybridisation signals (red) and four chromosomes had 18SrDNA hybridisation signals (green) (Fig. 7C), confirming that the sample was hexaploid material (2n = 6x = 42) with large-scale repetitive amplification (Table 7, Fig. 7D).

**Table 7.** Genome size and ploidy of *Elymusmultiramosus* compared with two germplasm of *Elymussibiricus* with sample numbers 15-262 and 16-118.

Germplasm	Reference	Fluorescence intensity of reference	Fluorescence intensity of germplasm	Ratio	Genome (Gb)	Ploidy
*Elymusmultiramosus*	Corn	64.26	263.87	4.11	9.44	6X
*Elymussibiricus* 15-262	Corn	63.26	163.04	2.58	5.93	4X
*Elymussibiricus* 16-118	Corn	63.66	170.26	2.67	6.15	4X

### ﻿Compound spike development

*Elymusmultiramosus* seeds (five inflorescences included about 80 seeds) were sown in May 2021 and, after the rejuvenation in April 2022, the development of compound spike was detected from more than 30 inflorescences.

**Figure 8.**
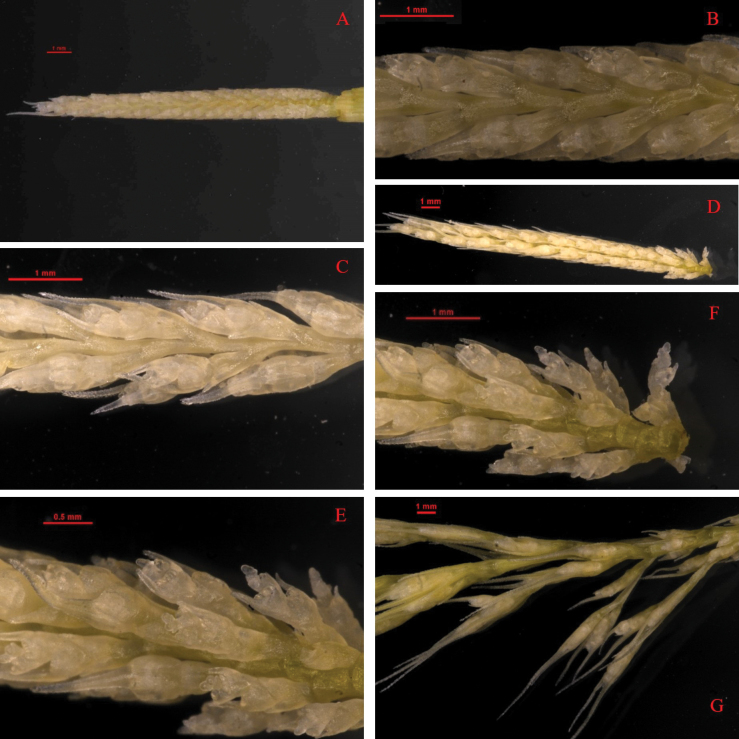
The compound spike development **A, B** are the stages of the initial floret **C** is the stage of the start of the spikelet protuberance on the branches **D–F** show the stage of branching starting from the base of the compound spike **G** is the stage of branches elongation and spikelet formation.

In the development of the compound spike of *Elymusmultiramosus*, its stages have been identified - the stages of the initial floret, the stage of the beginning of the spikelet protuberance on the branches, branching from the base of the compound spike and finally branches elongation and spikelet formation (Fig. 8A–G).

### ﻿Phylogenetic analysis

**Figure 9.**
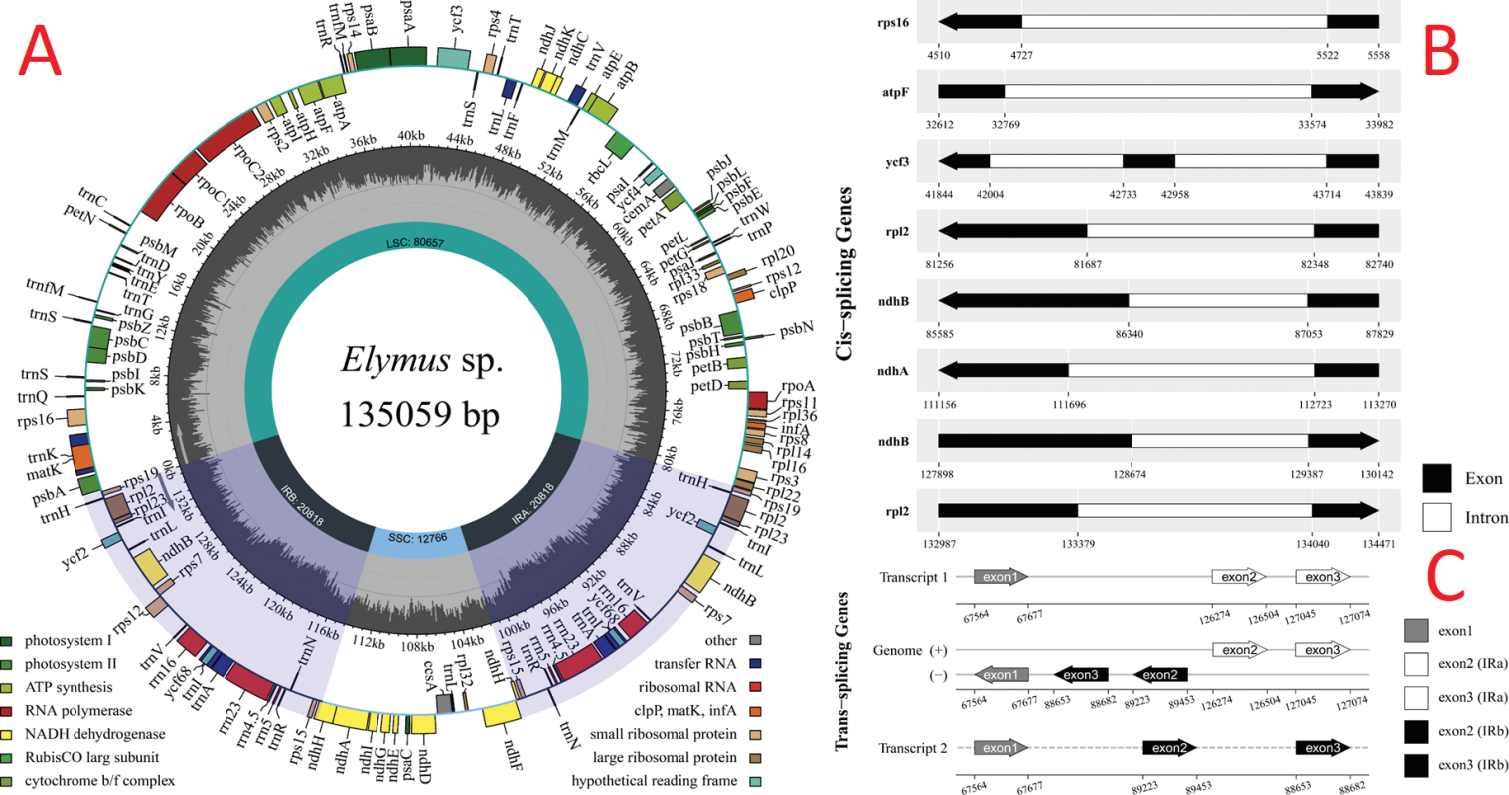
Schematic circular map of overall features of *E.multiramosus* chloroplast genome. Graphic showing features of its plastome was generated using CPGview. The map contains three tracks. From the inner circle, the first track depicts the sizes of the inverted repeats (IRa and IRb), small single-copy (SSC) and large single-copy (LSC). The second track plots the distribution of GC contents along the plastome. The third track displays the genes belonging to different functional groups with different coloured boxes. The outer and inner genes are transcribed in the clockwise and counter-clockwise directions, respectively (**A**). Cis-splicing gene distribution map (**B**). Trans-splicing gene distribution map (**C**).

The chloroplast genome of *Elymusmultiramosus* is 135,059 bp in length with an average sequencing depth of 112×h. It exhibits a typical four-level structure consisting of a large single-copy (LSC) region of 80,667 bp in length, a small single-copy (SSC) region of 12,766 bp in length and two inverted repeat regions (lRa/IRb) of 20,813 bp in length (Fig. 9A). The whole chloroplast genome has a CG content of 38.3% and encodes a total of 134 genes, including 88 protein-coding genes, 38 tRNA genes and eight rRNA genes. Amongst them, seven protein-coding genes (rps16, atpF, rpl2, ndhB, ndhA, ndhB, petB and rpl2) contained one intron and one gene ycf3 had two introns (Fig. 9B). In addition, the single 5′ end of the trans-spliced gene rps12 is located in the large single-copy region, whereas the duplicated 3′ end exons are located in the two trans-repeat regions (Fig. 9C).

**Figure 10.**
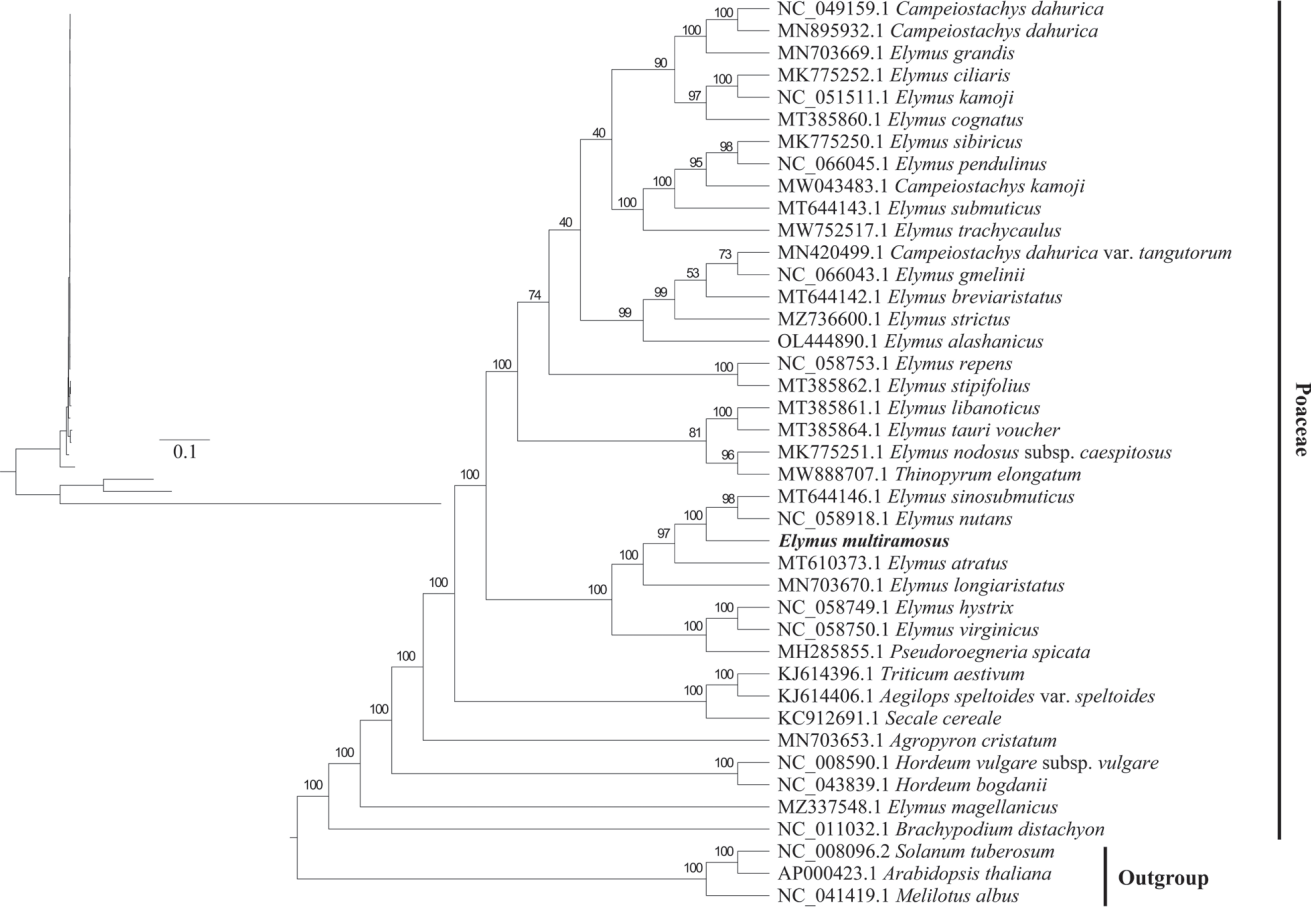
Phylogenomic reconstruction result of *E.multiramosus* with 1000 bootstraps in RAxML. *Solanumtuberosum*, *Arabidopsisthaliana* and *Melilotusalbus* are the outgroup.

In the molecular phylogeny, *Elymusmultiramosus* from Qinghai, north-western China, is phylogenetically positioned as a distinct lineage. The lineage comprising *Elymussinosubmuticus* from Sichuan, east of the Tibetan Plateau and *Elymusnutans* from the Himalayas forms a sister group to *Elymusmultiramosus*, suggesting that these three species share a common ancestor that is distinct from the lineage leading to *Elymusatratus* from Gansu, north of the Tibetan Plateau (Fig. 10). The final phylogenetic analysis revealed consistent evolutionary relationships, with results from both methods corroborating each other, thereby ensuring the credibility of the conclusions.

The new species is similar to *Elymusnutans*, a perennial herbaceous plant is primarily distributed in the temperate regions of Asia, especially common in the alpine meadows of the Tibetan Plateau and its surrounding areas in China. It can be easily distinguished from that species by its compound spike. With the molecular evidence, thus, we confirmed that it represented a new species discovered within the alpine regions. It is necessary to introduce the compound spike into *Elymus* L. for classification.

## ﻿Discussion

Inflorescences amongst the Triticeae tribe, exemplified by barley, likely evolved from an ancestral compound spike into the more typical unbranched spike observed today. Grass inflorescences, according to proposed evolutionary models, originated from a primitive ancestral form featuring a relatively small panicle-like branching system, comprising primary and secondary branches standing alone at nodes (Vegetti and Anton 1995). This ancestral structure is a compound spike, still evident in tribes like Oryzeae and Andropogoneae, which maintain complex compound shapes with true-lateral long primary and secondary branches. Conversely, other grasses, such as *Brachypodiumdistachyon*, exhibit reduced inflorescence complexity, characterised by smaller lateral pedicels that culminating in single multi-floweret spikelets (Kellogg et al. 2013; Remizowa et al. 2013).

It was found that the inflorescence differentiation of *Elymussibiricus* encompasses the initiation, elongation, single-ridged or double-ridged spikelet and flower differentiation stages (Mao et al. 2004). However, the development of the compound spike in *Elymusmultiramosus* passes through distinct stages. In the inflorescence differentiation of *E.multiramosus*, the base of the spikelet, located in the middle and lower part of the inflorescence axis, functions as an independent component of the entire inflorescence. It undergoes a specialised process of branch initiation and elongation, unlike the upper part of the inflorescence and related species, which do not exhibit this process. This process initiates with branching at the base of the compound spike, followed by branch elongation and subsequent spikelet formation. These stages are critical for understanding the reproductive and developmental intricacies of *E.multiramosus* within its genus. This process of compound spike formation is crucial for crop yield formation and the synchronous maturation of seeds (Wang et al. 2021).

The inflorescences of *Elymus* are described as racemes. In the Flora of China (Hua 2007), *Elymus* has been described as: the spike erect to nodding, spikelets 1 or 2(-4) per node, rarely very short pedicellate, appressed to rachis, clearly laterally compressed, usually all similar, with 2–10 or more florets. The spike is one type of the racemes. The compound spike has branches in the rhachis, each branch being like a mini spike, which is the key to the new species.

*Elymusmultiramosus* is characterised by distinctive morphological features, especially the glumes, which are shorter than the first floret and the awns, which are 9 to 12 mm long and exceed the lemma. The robust stature and longer inflorescences, along with the predominantly unilateral arrangement of spikelets, further distinguish *Elymusmultiramosus* from other species. These features are crucial for identifying and distinguishing *E.multiramosus* from closely-related species like *Elymusnutans*.

Moreover, *Elymusmultiramosus* is distinguished by its compound spike inflorescence, which is a key feature that distinguishes it from other *Elymus* species with simpler inflorescences. This compound spike is defined by a distinct main shoot axis from which lateral branches emerge, in contrast to simpler *Elymus* inflorescences where flowers form directly from the main axis (Benlloch et al. 2007). In *Elymusmultiramosus*, these lateral branches, or rhachillas, originate at the base of the main axis, extending into 3–6 mini-spike-like branches arranged distichously. Notably, the length of these branches increases progressively from the top towards the bottom of the spike, demonstrating a complex structural adaptation. These morphological differences underline the divergence between the species and are vital for identifying and differentiating *E.multiramosus* from its relatives.

As the world’s highest and youngest plateau, the Qinghai-Tibetan Plateau has had a profound impact on the phylogeny of *Elymus* species due to its unique geographical and climatic conditions. Notably, larger genome size variations occurred in mid-altitude populations (3900–4300 m) compared with populations at other altitudes, suggesting a distinct altitudinal pattern in genome size variation. This variation plays a crucial role in shaping genome evolution according to altitude and supports that mid-altitude regions serve as centres of genetic richness, facilitating species adaptation to highland environmental conditions and providing valuable germplasm for utilisation and conservation (Chen 2022b). *Elymus* species originated through a typical allopolyploidy process, involving the combination of different genomes. Cytological studies suggest that five basic genomes — St, Y, H, P and W — exist in various combinations across *Elymus* species. The evolutionary history of *Elymus* species on the Qinghai-Tibetan Plateau involve multiple origins due to the introduction of different H genome donors (Liu et al. 2006).

*Elymusmultiramosus* has only been found in a small area at the north-western Qinghai-Tibetan Plateau, growing in a dry, rocky area at an elevation of 3722 metres. Its restricted geographical distribution positions it as a distinct lineage in phylogenetic analyses. *Elymussinosubmuticus* and *Elymusnutans*, found in Sichuan and the Himalayas, respectively, form a sister group with *Elymusmultiramosus*, sharing a common ancestor, distinct from *Elymusatratus* from northern Gansu. This lineage divergence reflects the natural selection and adaptive evolution on *Elymusmultiramosus*. Furthermore, phylogenetic analyses have shown that *Elymus* species on the Qinghai-Tibetan Plateau have experienced multiple origins and gene flow events during their evolution (Yan et al. 2024). The uniqueness of this species reflects its independent evolutionary history in the specific ecological context of the north-western Qinghai-Tibetan Plateau.

Through detailed examination of morphological, phylogenetic and developmental characteristics, it is supported that *Elymusmultiramosus* is classified within the section Elymus. This comprehensive analysis supports its classification and provides insights into its evolutionary development and agronomic potential (Vegetti and Anton 1995). Further research, particularly in the domain of genetic diversity and environmental adaptation, would enrich our understanding of its role within its ecosystem and its agricultural value.

## Supplementary Material

XML Treatment for Elymus multiramosus

## ﻿Additional information

### Author ORCIDs

Yong-Chao Zhang https://orcid.org/0000-0003-4656-5608

Yan Qin https://orcid.org/0000-0002-5638-3472

### Data availability

All of the data that support the findings of this study are available in the main text or Supplementary Information.

## Supplementary materials

Supplementary material 1 Supplementary image Data type pdf https://binary.pensoft.net/file/1172930This dataset is made available under the Open Database License (http://opendatacommons.org/licenses/odbl/1.0/). The Open Database License (ODbL) is a license agreement intended to allow users to freely share, modify, and use this Dataset while maintaining this same freedom for others, provided that the original source and author(s) are credited.Yong-Chao Zhang, Xiao-Xing Wei, Yan Qin, Yong Liu, Shu-Zhen Zhang, Zhi-Feng Jia, Wen-Hui Liu
