# Supplementary figures and images for: ﻿Elymusmultiramosus (Poaceae), a new species from the north-western Qinghai-Tibetan Plateau, China
Source: PhytoKeys. 2024 Nov 12;249:51–73. doi: 10.3897/phytokeys.249.127632 (PMC11576837; doi:10.3897/phytokeys.249.127632)

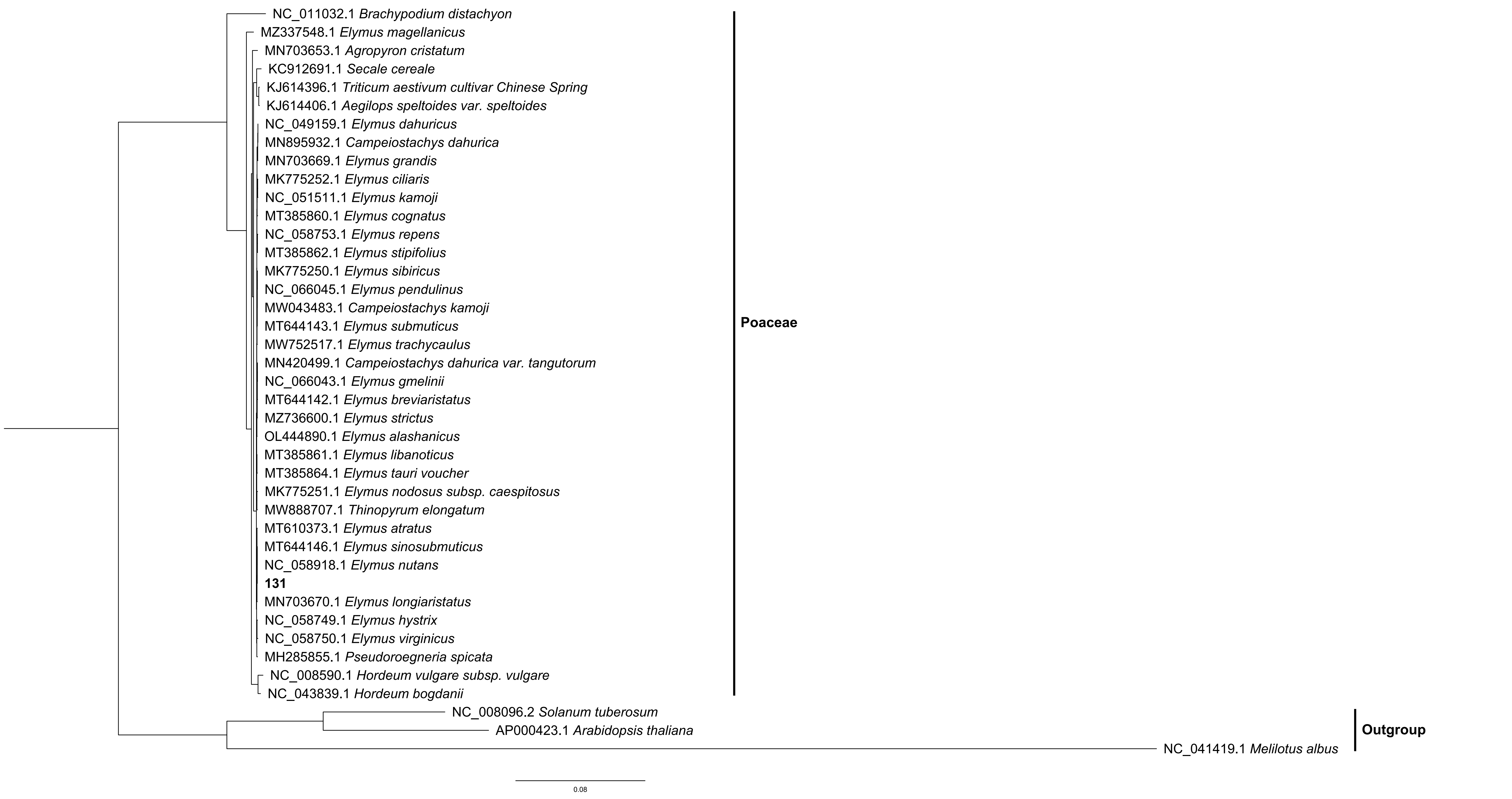

Supplement: Supplementary material 1 — Supplementary image [file phytokeys-249-051_article-127632__-s001.pdf]
